# Variation in symptoms of common mental disorders in the general population during the COVID-19 pandemic: longitudinal cohort study

**DOI:** 10.1192/bjo.2024.2

**Published:** 2024-02-12

**Authors:** Rob Saunders, Joshua E. J. Buckman, Jae Won Suh, Peter Fonagy, Stephen Pilling, Feifei Bu, Daisy Fancourt

**Affiliations:** CORE Data Lab, Centre for Outcomes Research and Effectiveness (CORE), Research Department of Clinical, Educational & Health Psychology, University College London, UK; CORE Data Lab, Centre for Outcomes Research and Effectiveness (CORE), Research Department of Clinical, Educational & Health Psychology, University College London, UK; and iCope Psychological Therapies Service, Camden & Islington NHS Foundation Trust, St Pancras Hospital, London, UK; Research Department of Clinical, Educational and Health Psychology, University College London, UK; CORE Data Lab, Centre for Outcomes Research and Effectiveness (CORE), Research Department of Clinical, Educational & Health Psychology, University College London, UK; and Camden & Islington NHS Foundation Trust, London, UK; Department of Behavioural Science and Health, University College London, UK

**Keywords:** Anxiety or fear-related disorders, depressive disorders, epidemiology, statistical methodology, cohort study

## Abstract

**Background:**

A significant rise in mental health disorders was expected during the COVID-19 pandemic. However, referrals to mental health services dropped for several months before rising to pre-pandemic levels.

**Aims:**

To identify trajectories of incidence and risk factors for common mental disorders among the general population during 14 months of the COVID-19 pandemic, to inform potential mental health service needs.

**Method:**

A cohort of 33 703 adults in England in the University College London COVID-19 Social Study provided data from March 2020 to May 2021. Growth mixture modelling was used to identify trajectories based on the probability of participants reporting symptoms of depression (Patient Health Questionnaire-9) or anxiety (Generalised Anxiety Disorder-7) in the clinical range, for each month. Sociodemographic and personality-related characteristics associated with each trajectory class were explored.

**Results:**

Five trajectory classes were identified for depression and anxiety. Participants in the largest class (62%) were very unlikely to report clinically significant symptom levels. Other trajectories represented participants with a high likelihood of clinically significant symptoms throughout, early clinically significant symptoms that reduced over time, clinically significant symptoms that emerged as the pandemic unfolded and a moderate likelihood of clinically significant symptoms throughout. Females, younger adults, carers, those with existing mental health diagnoses, those that socialised frequently pre-pandemic and those with higher neuroticism scores were more likely to experience depression or anxiety.

**Conclusions:**

Nearly 40% of participants followed trajectories indicating risk of clinically significant symptoms of depression or anxiety. The identified risk factors could inform public health interventions to target individuals at risk in future health emergencies.

The COVID-19 pandemic was anticipated to lead to a global ‘tsunami’ of mental health disorders,^1^ resulting from months of fear about the virus, the impact on relationships, loss of friends and family, loss of income or employment, post-viral effects on health and the psychological effects of stay-at-home orders.^2^ Approximately 30% of adults appear to have experienced clinically significant symptoms (scoring above established thresholds on validated measures) of depression or anxiety, referred to as common mental disorders (CMDs) in the UK and USA, but this decreased after the initial lockdown restrictions eased.^3–5^ Similarly, there was an initial increase in symptoms in mental health service attendees on average in the UK, but the effect was not maintained during the period of ‘lockdown’,^6^ and although antidepressant prescribing increased, this appears to be in line with trends from recent years rather than an indication of a step-change associated with the pandemic.^7^ As such, the anticipated ‘tsunami’ of mental health disorders is not apparent at the general population level. Yet, it is likely that mental health in some groups of people was disproportionately negatively affected by the pandemic, and it is important to identify these groups to better plan for future health emergencies. Studies conducted in different countries have consistently found heterogeneous trajectories of mental health symptoms during the pandemic, demonstrating that distinct subpopulations with elevated CMDs did not recover after the initial months of the pandemic.^4,5,8–10^ Factors associated with such negative symptom trajectories include younger age, female gender, lower income and a previous mental health diagnosis.^4,11,12^ However, few studies have investigated differential CMD symptom trajectories beyond the first 6 months of the pandemic,^13–15^ and most have analysed changes in CMD symptom measure scores regardless of clinical significance. To assist mental health services in planning for a potential future surge event in a health emergency like the COVID-19 pandemic, it is important to model the trajectories of clinical need in the population rather than change in symptom scores. To better understand the potential need for clinical interventions for CMDs and how these have changed over the course of the COVID-19 pandemic, a focus on modelling levels of CMD symptoms that are likely to be clinically significant and data spanning a wider time interval is needed. The present study aimed to identify statistically distinct trajectories of clinically significant CMD symptoms and the associated characteristics of participants during the first 14 months of the pandemic (March 2020 to May 2021), across three national lockdowns in England.

## Method

### Participants

The analytic sample was drawn from the COVID-19 Social Study, a large panel study of the psychological and social experiences of over 70 000 adults (age ≥18 years) in the UK during the COVID-19 pandemic.^16^ The study has been described in detail elsewhere^17,18^ and further information is provided in Supplementary Appendix A, available at https://doi.org/10.1192/bjo.2024.2. Participants were recruited using three main approaches: (a) convenience sampling, such as advertisement through social media and mailing lists; (b) targeted recruitment of people who were from a low-income background, had no or few educational qualifications or were unemployed; and (c) promotion to vulnerable groups (e.g. those with pre-existing mental health conditions, older adults, carers and people experiencing domestic violence or abuse).

The present study included participants residing in England, recruited between 21 March 2020 and 6 September 2020. As recruitment to the study was not random, the sample was weighted by the proportions of gender, age, ethnicity and education in England, obtained from the Office for National Statistics.^19^ From a total of 58 485 participants with available data, 21 051 did not provide data for at least three time points and were therefore excluded. Of the remaining individuals, 3731 people with missing data on predictors including variables for weighting were additionally excluded, resulting in a study sample of 33 703 participants (flow diagram presented in Supplementary Fig. 1). All participants gave written consent when they signed up to the study. The study was approved by the University College London Research Ethics Committee (approval number 12467/005).

### Measures

At recruitment, participants were asked to self-report their sociodemographic characteristics and previous mental health diagnoses via an online survey. The following factors were self-reported: age, gender, ethnicity, income, educational attainment, living arrangements with others, overcrowding (i.e. fewer than one room per person in household), history of mental health condition (yes or no) or chronic physical health diagnoses (yes or no), pre-pandemic amount of social contact, keyworker status (e.g. health and social care worker) and personality (Big Five Inventory).^20^

Participants were asked to complete measures assessing symptoms of depression (Patient Health Questionnaire-9 (PHQ-9))^21^ and anxiety (Generalised Anxiety Disorder-7 (GAD-7))^22^ at recruitment and at each follow-up via the same online survey application. Follow-up data collection was initially conducted each week, with participants receiving an automatic email invitation to the next wave of data collection 7 days after completing each survey. Reminders were sent 24 and 48 h following the first automatic invitation, and if participants did not complete the survey after these reminders, they stopped receiving further invitations for follow-up. After 22 weeks, follow-up moved to monthly intervals in August 2021.^16^ At this point, individuals who had been lost to follow-up but did not formally unsubscribe from the study were invited to participate again. From then, automatic invitations for follow-up data collection were sent 28 days after completion of each survey. Retention rate at follow-up surveys was high throughout the pandemic (see Supplementary Appendix B).

The thresholds to indicate clinically significant levels of depression or anxiety symptoms used for the current study were ≥10 on the PHQ-9 and ≥8 on the GAD-7, in line with those used by English national psychological treatment services^23^ and the scale developers.^21^ Clinical caseness refers to symptom levels likely to meet the diagnostic criteria for the measured disorder. Throughout this study, we used the term ‘clinically significant’ to describe self-reported symptoms of depression or anxiety above the threshold of clinical caseness on the PHQ-9 or GAD-7. However, we acknowledge there is evidence that self-report measures have been found to overestimate prevalence of CMDs compared with diagnostic interviews.^24^ For further details on measures, participant characteristics and retention rate see Supplementary Appendix B and Supplementary Table 1.

### Analysis

Growth mixture modelling (GMM) was used to identify different trajectories of the incidence of reporting symptoms of depression and anxiety that were likely to be clinically significant, according to validated thresholds. Depression and anxiety scores were dichotomised using the thresholds listed above to create a binary variable indicating the presence or absence of symptoms likely to be in the clinically significant range. This information is likely to be more relevant than changes in symptom scores for future health service organisation and care planning, as it indicates the number of people with degrees of symptom severity more likely to require clinical care. GMM was used to identify statistically distinct subgroups of individuals based on their patterns of change in the likelihood of reporting clinically significant symptoms of depression or anxiety.^25,26^ Although GMM can be specified with pre-assumed forms of change, we made no prior assumption for the current analysis, instead leaving these to be determined by the data (by setting time scores as free parameters, achieved by specifying the first two time scores to 0 and 1 to allow model identification, and all others set to free parameters [*]).^17^ The following model fit statistics were considered for the GMM: the Vuong-Lo-Mendell-Rubin likelihood ratio test,^27^ the Akaike Information Criterion, the Bayesian Information Criterion and entropy values. Further information on the GMMs and model selection decisions is presented in Supplementary Appendix C. Full information maximum likelihood through the expectation–maximisation algorithm^28^ was used to handle missing data in the GMMs, and survey weights were trimmed at the top 90% to minimise the impact of extreme weights.^4,29^ GMM analyses were conducted in Mplus for Windows, version 8 (Muthén & Muthén, Los Angeles, USA; see www.statmodel.com).^30^

Following the identification of depression and the anxiety trajectories, multinomial logistic regression models were constructed to explore associations between baseline participant characteristics and trajectory class. We used characteristics that have been associated with increased risk of clinically significant symptoms during the COVID-19 pandemic in previous studies, including age, gender, income, education, living situation, previous mental or physical health conditions, keyworker status, personality characteristics and the reported amount of social contact before the pandemic.

## Results

### Descriptive statistics

A total of 33 703 participants met inclusion criteria, with descriptive statistics presented in Table 1. There was an overrepresentation of women (76%) and individuals with university-level degrees (70%). There was an underrepresentation of individuals from minority ethnic groups (5%) compared with the general population. We applied weights that better represented the general population. The weighted sample consisted of 20 490 (61%) women, 2777 (8%) individuals from minority ethnic groups and 1650 (49%) individuals with university-level degrees. A previous mental health diagnosis at the point of study entry was reported by 6880 (20%) individuals.

**Table 1:** Descriptive statistics

	Raw data		Weighted data	
	*n*	%	*n*	%
Gender				
Women	25 567	76	20 490	61
Men	8136	24	13 213	39
Age in years				
18–29	2182	6	3856	12
30–45	9134	27	12 158	36
46–59	11 364	34	11 621	34
≥60	11 023	33	6038	18
Ethnicity				
White	32 075	95	30 926	92
Minority ethnic groups	1628	5	2777	8
Household income				
<£30 000	12 557	37	13 189.49	39
≥£30 000	21 146	63	20 514	61
Keyworker				
No	25 995	77	25 390	75
Yes	7708	23	8313	25
Highest level of educational qualifications				
General Certificate of Secondary Education (GCSE) or below	4378	13	8058	24
A-levels or equivalent	5660	17	9175	27
Undergraduate degree or above	23 665	70	1650	49
Carer				
No	28 515	85	28 767	85
Yes	5188	15	4936	15
Living situation				
Alone	6841	20	5845	17
With others, without children	18 150	54	17 672	52
With others, including children	8712	26	10 186	30
Overcrowded				
No	30 382	90	29 005	86
Yes	3321	10	4698	14
Urban/rural				
Rural	7095	21	6456	19
Urban	26 608	79	27 247	81
Diagnosed mental illness				
No	27 686	82	26 823	80
Yes	6017	18	6880	20
Long-term physical health condition				
No	20 213	60	21 047	62
Yes	13 490	40	12 656	38
Previous social contact frequency				
Every day	3509	10	3181	9
Three or more times a week	8642	26	7471	22
Once or twice a week	11 768	35	11 903	35
Once or twice a month	6372	19	6950	21
Less than once a month	3412	10	4197	12
Big Five personality factor	Mean	s.d.	Mean	s.d.
Neuroticism	11.29	4.28	11.63	4.40
Extraversion	12.91	4.29	12.56	4.31
Openness	15.45	3.28	15.04	3.33
Agreeableness	15.55	3.06	15.46	3.12
Conscientiousness	15.91	2.95	15.72	2.99

### Trajectories of depression and anxiety symptom change

**Fig. 1:**
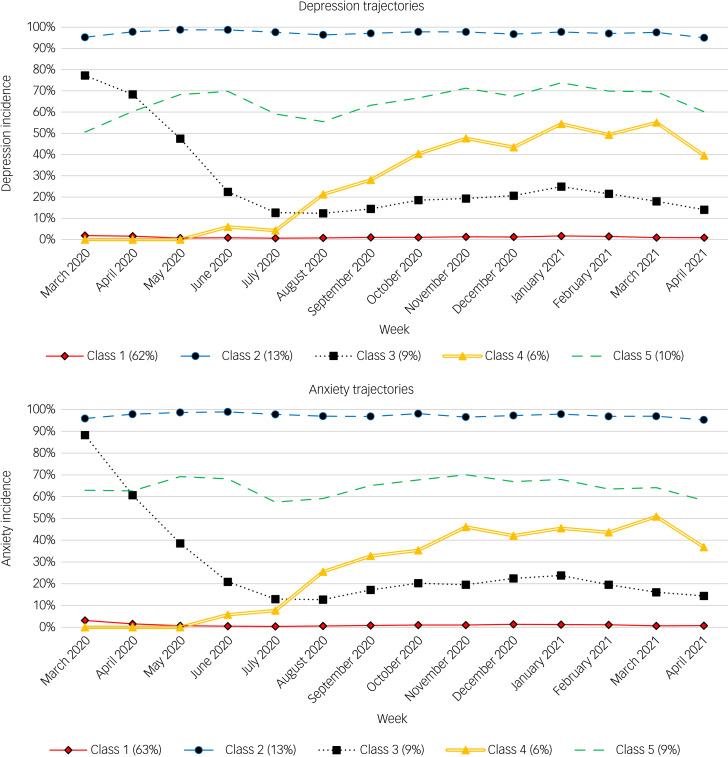
Identified trajectories for depression (top panel) and anxiety (bottom panel) symptom incidence.

GMM was performed on both the GAD-7 and PHQ-9 data independently. A total of 321 333 PHQ-9 scores (indicating clinically significant symptoms *n* = 60 872[19%]) and 321 333 GAD-7 scores (indicating clinically significant symptoms *N* = 59 085[18%]) were analysed. For both depression and anxiety symptoms, the five-class solution appeared to be the best fitting (see Supplementary Table 2 for fit statistics). The trajectories (in Fig. 1) were similar between measures. The classes are described as follows: Class 1, low likelihood of clinically significant symptoms of depression or anxiety throughout the study period; Class 2, high likelihood of clinically significant symptoms throughout the study period; Class 3, clinically significant symptoms early in the pandemic, which reduced within the early months; Class 4, clinically significant symptoms that emerged after 5 months of the pandemic; and Class 5, moderate likelihood of incident depression or anxiety throughout the study period.

Class 1 was the largest trajectory class, indicating people who were extremely unlikely to report levels of depression (62% of the total sample) or anxiety (63% of the total sample) that were likely to be clinically significant throughout the pandemic period studied. Nearly 40% of people were members of the four other trajectory classes, which reported clinically significant levels of depression or anxiety at least once during the first 14 months of the COVID-19 pandemic. In particular, 13% of people belonged to Class 2, meaning they consistently reported levels of depression or anxiety likely to be clinically significant throughout the observation period. Other groups reported a moderate likelihood of clinically significant symptoms of depression or anxiety for several months during the pandemic. Class 4 was the only group that appeared to show an increase in the likelihood of clinically significant mental health symptoms in the lead up to the November 2020 lockdown in the UK, representing 6% of the total sample. Supplementary Appendix D presents correspondence between depression and anxiety classes (i.e. the proportion of individuals across to two sets of classes), and it was observed that the levels of similarity when individuals were in the same depression and anxiety class was higher for classes 1 and 2, with less correspondence for the other classes (Supplementary Table 4).

### Associations with trajectory classes

Descriptive statistics of each identified trajectory class are presented in Supplementary Table 5. Sociodemographic and personality characteristics independently associated with the likelihood of belonging to depression trajectory classes and anxiety trajectory classes are presented in Tables 2 and 3, respectively. Class 1 (low incidence) is used as the reference class in these multinomial regression models. Further logistic regression analyses were performed comparing Class 2 and Class 3 for both the anxiety and depression trajectories, given the similar proportion of individuals in the clinical range at the start of the study period for these two classes (presented in Supplementary Table 6). Classes 4 and 5 were then compared because of the similar final proportion of individuals in each class observed to be scoring in the clinical range in the last available data in the study period (presented in Supplementary Table 7).

**Table 2:** Participant characteristics associated with depression trajectory classes

	Class 2 (versus class 1)			Class 3 (versus class 1)			Class 4 (versus class 1)			Class 5 (versus class 1)		
	RRR	95% CI	*P*-value	RRR	95% CI	*P*-value	RRR	95% CI	*P*-value	RRR	95% CI	*P*-value
Gender: women (versus men)	**1.12**	**(1.02–1.22)**	**0.014**	**1.44**	**(1.31–1.58)**	**<0.001**	**1.36**	**(1.23–1.51)**	**<0.001**	**1.34**	**(1.23–1.47)**	**<0.001**
Age: 18–29 years (versus ≥60 years)	**3.38**	**(2.84–4.01)**	**<0.001**	**3.43**	**(2.85–4.14)**	**<0.001**	**1.68**	**(1.37–2.06)**	**<0.001**	**2.31**	**(1.94–2.75)**	**<0.001**
Age: 30–45 years (versus ≥60 years)	**2.52**	**(2.18–2.92)**	**<0.001**	**2.44**	**(2.07–2.88)**	**<0.001**	**1.73**	**(1.46–2.03)**	**<0.001**	**1.98**	**(1.70–2.29)**	**<0.001**
Age: 46–59 years (versus ≥60 years)	**1.88**	**(1.64–2.15)**	**<0.001**	**2.01**	**(1.73–2.35)**	**<0.001**	**1.37**	**(1.18–1.59)**	**<0.001**	**1.50**	**(1.31–1.72)**	**<0.001**
Ethnicity: minority ethnic (versus White)	**1.43**	**(1.25–1.64)**	**<0.001**	1.13	(0.98–1.30)	0.102	0.97	(0.82–1.15)	0.751	**1.28**	**(1.12–1.47)**	**<0.001**
Education: low (versus high)	**1.51**	**(1.35–1.69)**	**<0.001**	**1.24**	**(1.10–1.40)**	**<0.001**	1.11	(0.97–1.26)	0.138	1.09	(0.97–1.22)	0.156
Education: medium (versus high)	**1.41**	**(1.28–1.55)**	**<0.001**	**1.12**	**(1.02–1.24)**	**0.023**	1.01	(0.90–1.13)	0.892	0.99	(0.90–1.10)	0.915
Income: <£30 000 (versus ≥£30 000)	**1.96**	**(1.80–2.14)**	**<0.001**	**1.27**	**(1.16–1.39)**	**<0.001**	**1.26**	**(1.13–1.40)**	**<0.001**	**1.45**	**(1.32–1.58)**	**<0.001**
Living alone (versus with others, no children)	**1.59**	**(1.43–1.77)**	**<0.001**	**1.29**	**(1.14–1.45)**	**<0.001**	1.06	(0.93–1.21)	0.372	**1.18**	**(1.05–1.32)**	**0.004**
Living with others, with children (versus with others, no children)	**1.14**	**(1.04–1.26)**	**0.005**	**1.26**	**(1.14–1.38)**	**<0.001**	1.05	(0.94–1.17)	0.394	0.96	(0.87–1.06)	**0.441**
Mental health diagnosis (versus none)	**6.15**	**(5.64–6.71)**	**<0.001**	**2.85**	**(2.59–3.15)**	**<0.001**	**1.90**	**(1.68–2.16)**	**<0.001**	**3.49**	**(3.18–3.84)**	**<0.001**
Carer (versus not a carer)	**1.43**	**(1.29–1.59)**	**<0.001**	**1.22**	**(1.09–1.36)**	**0.001**	**1.26**	**(1.12–1.43)**	**<0.001**	**1.19**	**(1.06–1.33)**	**0.002**
Keyworker (versus not a keyworker)	0.97	(0.88–1.06)	0.470	**1.18**	**(1.07–1.29)**	**0.001**	1.07	(0.97–1.19)	0.191	**1.12**	**(1.02–1.23)**	**0.015**
Long-term health condition (versus none)	**2.26**	**(2.08–2.45)**	**<0.001**	**1.42**	**(1.30–1.54)**	**<0.001**	**1.42**	**(1.29–1.56)**	**<0.001**	**1.62**	**(1.49–1.76)**	**<0.001**
Overcrowded living (versus not)	**1.38**	**(1.24–1.54)**	**<0.001**	**1.43**	**(1.29–1.60)**	**<0.001**	1.12	(0.98–1.28)	0.108	**1.24**	**(1.11–1.39)**	**<0.001**
Rural (versus urban)	0.96	(0.87–1.06)	0.433	1.08	(0.97–1.19)	0.166	1.01	(0.90–1.14)	0.832	**0.87**	**(0.78–0.97)**	**0.009**
Social: every day (versus once/twice a week)	**1.43**	**(1.24–1.65)**	**<0.001**	**1.16**	**(1.01–1.34)**	**0.040**	**1.21**	**(1.02–1.42)**	**0.025**	**1.18**	**(1.02–1.36)**	**0.026**
Social: three/four times a week (versus once/twice a week)	1.02	(0.91–1.14)	0.736	1.01	(0.91–1.13)	0.838	1.07	(0.95–1.21)	0.289	0.95	(0.85–1.06)	0.352
Social: once/twice a month (versus once/twice a week)	**1.17**	**(1.05–1.31)**	**0.004**	0.95	(0.85–1.06)	0.380	**1.18**	**(1.04–1.33)**	**0.008**	1.09	(0.98–1.22)	0.107
Social: less once month (versus once/twice a week)	**1.76**	**(1.56–1.98)**	**<0.001**	1.05	(0.91–1.21)	0.485	1.09	(0.93–1.27)	0.308	**1.38**	**(1.21–1.56)**	**<0.001**
Personality: neuroticism	**1.23**	**(1.22–1.24)**	**<0.001**	**1.16**	**(1.14–1.17)**	**<0.001**	**1.07**	**(1.06–1.08)**	**<0.001**	**1.15**	**(1.13–1.16)**	**<0.001**
Personality: extraversion	**0.97**	**(0.96–0.98)**	**<0.001**	1.00	(0.99–1.01)	0.537	0.99	(0.98–1.00)	0.197	0.99	(0.98–1.00)	0.195
Personality: openness	**1.06**	**(1.05–1.07)**	**<0.001**	**1.05**	**(1.04–1.06)**	**<0.001**	**1.04**	**(1.03–1.06)**	**<0.001**	**1.06**	**(1.05–1.07)**	**<0.001**
Personality: agreeableness	0.99	(0.98–1.01)	0.246	0.99	(0.98–1.01)	0.248	0.99	(0.98–1.01)	0.503	0.99	(0.98–1.00)	0.084
Personality: conscientiousness	**0.94**	**(0.93–0.95)**	**<0.001**	**0.95**	**(0.93–0.96)**	**<0.001**	**0.97**	**(0.95–0.98)**	**<0.001**	**0.95**	**(0.93–0.96)**	**<0.001**

Numbers in bold are statistically significant at *P* < 0.05. RRR, relative risk ratio.

**Table 3:** Participant characteristics associated with anxiety trajectory classes

	Class 2 (versus class 1)			Class 3 (versus class 1)			Class 4 (versus class 1)			Class 5 (versus class 1)		
	RRR	95% CI	*P*-value	RRR	95% CI	*P*-value	RRR	95% CI	*P*-value	RRR	95% CI	*P*-value
Gender: women (versus men)	1.03	(0.94–1.13)	0.570	**1.46**	**(1.32–1.61)**	**<0.001**	**1.48**	**(1.33–1.64)**	**<0.001**	**1.34**	**(1.22–1.47)**	**<0.001**
Age: 18–29 years (versus ≥60 years)	**3.47**	**(2.89–4.15)**	**<0.001**	**2.99**	**(2.47–3.61)**	**<0.001**	**1.80**	**(1.47–2.2)**	**<0.001**	**2.79**	**(2.33–3.35)**	**<0.001**
Age: 30–45 years (versus ≥60 years)	**2.75**	**(2.35–3.22)**	**<0.001**	**2.41**	**(2.04–2.85)**	**<0.001**	**1.71**	**(1.45–2.02)**	**<0.001**	**2.10**	**(1.79–2.45)**	**<0.001**
Age: 46–59 years (versus ≥60 years)	**1.75**	**(1.51–2.02)**	**<0.001**	**1.80**	**(1.54–2.11)**	**<0.001**	**1.35**	**(1.15–1.57)**	**<0.001**	**1.49**	**(1.28–1.73)**	**<0.001**
Ethnicity: minority ethnic (versus White)	**1.35**	**(1.17–1.55)**	**<0.001**	1.12	(0.96–1.29)	0.139	**1.22**	**(1.05–1.43)**	**0.012**	**1.41**	**(1.23–1.61)**	**<0.001**
Education: low (versus high)	**1.25**	**(1.11–1.41)**	**<0.001**	1.09	(0.96–1.22)	0.180	**0.81**	**(0.71–0.94)**	**0.004**	1.06	(0.94–1.19)	0.353
Education: medium (versus high)	**1.11**	**(1.01–1.23)**	**0.040**	0.93	(0.84–1.03)	0.172	0.97	(0.86–1.08)	0.567	0.93	(0.84–1.03)	0.170
Income: <£30 000 (versus ≥£30 000)	**1.62**	**(1.48–1.77)**	**<0.001**	**1.11**	**(1.01–1.22)**	**0.026**	**1.18**	**(1.06–1.31)**	**0.003**	**1.29**	**(1.17–1.41)**	**<0.001**
Living alone (versus living with others, no children)	1.10	(0.98–1.23)	0.096	0.99	(0.87–1.11)	0.814	0.97	(0.85–1.11)	0.642	0.94	(0.83–1.05)	0.275
Living with others, with children (versus living with others, no children)	**1.11**	**(1.01–1.22)**	**0.039**	**1.21**	**(1.10–1.34)**	**<0.001**	0.96	(0.86–1.08)	0.515	**1.13**	**(1.03–1.25)**	**0.012**
Mental health diagnosis (versus none)	**4.96**	**(4.54–5.43)**	**<0.001**	**2.40**	**(2.18–2.66)**	**<0.001**	**2.02**	**(1.79–2.29)**	**<0.001**	**3.29**	**(3.00–3.62)**	**<0.001**
Carer (versus not a carer)	**1.37**	**(1.23–1.53)**	**<0.001**	**1.27**	**(1.13–1.41)**	**<0.001**	1.03	(0.90–1.17)	0.702	**1.20**	**(1.07–1.34)**	**0.002**
Keyworker (versus not a keyworker)	**0.98**	**(0.89–1.08)**	**0.629**	**1.26**	**(1.15–1.38)**	**<0.001**	1.04	(0.93–1.15)	0.513	**1.07**	**(0.98–1.18)**	**0.145**
Long-term health condition (versus none)	**1.81**	**(1.66–1.97)**	**<0.001**	**1.37**	**(1.26–1.50)**	**<0.001**	**1.35**	**(1.22–1.49)**	**<0.001**	**1.52**	**(1.39–1.65)**	**<0.001**
Overcrowded living (versus not)	**1.46**	**(1.31–1.63)**	**<0.001**	**1.26**	**(1.12–1.41)**	**<0.001**	**1.19**	**(1.04–1.36)**	**0.011**	**1.28**	**(1.14–1.43)**	**<0.001**
Rural (versus urban)	0.98	(0.88–1.09)	0.734	0.97	(0.87–1.08)	0.559	0.90	(0.79–1.01)	0.076	0.95	(0.85–1.05)	0.322
Social: every day (versus once/twice a week)	**1.43**	**(1.24–1.66)**	**<0.001**	**1.32**	**(1.14–1.52)**	**<0.001**	1.05	(0.88–1.25)	0.587	**1.26**	**(1.09–1.46)**	**0.002**
Social: three/four times a week (versus once/twice a week)	1.08	(0.97–1.21)	0.173	1.07	(0.96–1.20)	0.205	1.00	(0.88–1.13)	0.943	0.99	(0.88–1.11)	0.840
Social: once/twice a month (versus once/twice a week)	1.05	(0.94–1.17)	0.416	0.98	(0.88–1.10)	0.764	1.07	(0.94–1.21)	0.299	1.07	(0.95–1.19)	0.255
Social: less once month (versus once/twice a week)	**1.53**	**(1.35–1.73)**	**<0.001**	1.13	(0.98–1.29)	0.096	**1.25**	**(1.07–1.46)**	**0.004**	1.11	(0.97–1.28)	0.122
Personality: neuroticism	**1.38**	**(1.37–1.40)**	**<0.001**	**1.25**	**(1.23–1.26)**	**<0.001**	**1.09**	**(1.07–1.10)**	**<0.001**	**1.25**	**(1.23–1.26)**	**<0.001**
Personality: extraversion	1.00	(0.99–1.01)	0.395	**1.02**	**(1.01–1.03)**	**0.001**	1.01	(0.99–1.02)	0.269	1.00	(0.99–1.01)	0.589
Personality: openness	**1.08**	**(1.07–1.10)**	**<0.001**	**1.06**	**(1.05–1.08)**	**<0.001**	**1.02**	**(1.01–1.04)**	**0.001**	**1.08**	**(1.07–1.09)**	**<0.001**
Personality: agreeableness	**0.98**	**(0.97–0.99)**	**0.001**	0.99	(0.98–1.01)	0.394	1.00	(0.98–1.01)	0.841	0.99	(0.98–1.01)	0.304
Personality: conscientiousness	1.01	(1.00–1.02)	0.154	**1.02**	**(1.00–1.03)**	**0.034**	0.99	(0.97–1.00)	0.129	1.00	(0.99–1.02)	0.533

Numbers in bold are statistically significant at *P* < 0.05. RRR, relative risk ratio.

### Depression trajectories

Class 1 (low incidence) were older than the other classes, and had a higher ratio of men compared with women (Table 2). Being from a minority ethnic group was associated with following a Class 2 (high incidence) or Class 5 (moderate incidence) trajectory compared with Class 1. Previous mental health diagnoses were very prevalent in Class 2, but all classes were more likely to report previous mental health conditions, as well as physical health conditions, compared with Class 1. Being on a low income and being a carer were also independently associated with increased likelihood of following all trajectories, compared with Class 1. Differences in personality characteristics were also observed: higher neuroticism and openness, as well as lower conscientiousness, scores were associated with a greater likelihood of following trajectories for classes 2–5 compared with Class 1. Being a keyworker was associated with an increased likelihood of following trajectories for Class 3 (early clinically significant symptoms) and Class 5. Socialising every day before the pandemic was associated with increased likelihood of following all trajectories (compared with Class 1), whereas socialising less than once a month was also associated with following Class 2 and Class 5 trajectories. Living alone was associated with following all trajectory classes except Class 4 (emergent clinically significant symptoms).

Further analyses (presented in Supplementary Table 6) exploring the characteristics associated with Class 3 (early clinically significant symptoms) versus Class 2 (high incidence) trajectories, which had similar rates of clinically significant symptoms initially, identified that being a woman, a keyworker and having higher extraversion scores were associated with a greater likelihood of being in Class 3. Being from a minority ethnic group, lower levels of education, lower income, having a previous mental health diagnosis, being a carer, having higher neuroticism scores and having a long-term physical health condition were all associated with a decreased likelihood of being in Class 3. Supplementary Table 7 presents the comparison of factors associated with being a member of Class 5 (moderate clinically significant symptoms) versus Class 4 (emergent clinically significant symptoms). It was found that being younger, having a previous mental health diagnosis, having a long-term physical health condition, socialising less than once a month, having higher neuroticism and openness scores, and having lower conscientiousness scores were associated with a greater likelihood of being in Class 5.

### Anxiety trajectories

There were many similarities in the characteristics associated with anxiety trajectory classes 2–5 compared with Class 1 (Table 3). Younger age, previous mental and physical health conditions, lower income, overcrowded living conditions and higher openness and neuroticism scores were all associated with increased odds of following trajectory classes 2–5, indicating potentially clinically significant symptoms of anxiety at some point in the pandemic. Exceptions were that female gender was not associated with higher odds of being in Class 2 (high incidence), minoritized ethnic background was not associated with Class 3 and socialising every day or being a carer were not associated with Class 4 – these characteristics were each linked with higher likelihood of membership to all other classes (versus Class 1).

In further analyses comparing the characteristics of participants in classes 3 and 2, being a woman, having a higher income, not having a previous mental health diagnosis or long-term physical health condition, and socialising once or twice a week (versus less than once a month) was associated with greater odds of being in Class 3 (early clinically significant symptoms). Higher extraversion, lower neuroticism and being a keyworker (versus not) were also associated with an increased likelihood of being in Class 3 compared with Class 2. Finally, Class 4 (emergent clinically significant symptoms) and Class 5 (moderate clinically significant symptoms) were compared (see Supplementary Table 7). Individuals in Class 5 were more likely to be younger, have lower education, have a previous mental health diagnosis, be a carer, socialise more frequently and have higher neuroticism and openness scores compared with those in Class 4.

## Discussion

In this study, we observed that nearly four in ten people were members of trajectory classes that reported symptoms of depression or anxiety likely to be in the clinically significant range at least once during the first 14 months of the COVID-19 pandemic in England. However, there were changing patterns of incidence, with <20% of total individual PHQ-9 and GAD-7 scores during the study period likely to be above clinical levels. The majority of participants were members of Class 1 and extremely unlikely to report clinical levels of depression or anxiety (62 and 63% of the total sample, respectively); conversely, 13% of people (Class 2) were likely to report clinical levels of depression or anxiety throughout the observation period. Other groups reported a moderate likelihood of incident depression or anxiety for several months during the pandemic. Class 4 were the only group who appeared to show an increase in the likelihood of clinically significant mental health symptoms in the lead up to the November 2020 lockdown in the UK, but it represented just 6% of the sample. Sociodemographic factors, such as younger age, having previously been diagnosed with a mental or physical health condition, and having a low income, were independently associated with being in trajectory classes with greater likelihood of reporting depression or anxiety symptoms in the clinical range. In addition, higher neuroticism and openness scores, and socialising on a daily basis before the pandemic, were associated with an increased likelihood of following trajectories at greater risk of clinically significant depression and anxiety scores throughout the study period.

These findings confirm heterogeneous mental health trajectories identified earlier in the pandemic.^4,5^ Four of the five trajectories identified here (all except Class 4) are similar to ones observed in studies conducted with data from the first 6 months of the pandemic,^4,31^ as well as two studies that used data spanning the first 12 months of the pandemic.^14,15^ Class 4, representing a group of adults whose clinically significant symptoms emerged during the course of the pandemic, appears to be a particularly at-risk group regarding mental health. This trajectory class is somewhat similar to a group labelled as those with ‘deteriorating’ mental health, identified in a smaller study conducted over the first 12 months of the pandemic that used less frequent measurement of symptoms with the PHQ-ADS (Patient Health Questionnaire-Anxiety Depression Scale).^13^ The study found that having a history of mental health treatment, higher levels of loneliness and death anxiety, and lower levels of resilience were associated with a ‘deteriorating’ trajectory class compared with the ‘resilient’ class (corresponding to Class 1 in our study). Here, we found that individuals from Class 4 were, on average, younger than participants in Class 1, with higher levels of education but lower incomes, suggesting that perhaps working-age adults who were more likely to have insecure employment or to be on zero-hours contracts comprised this group, including young adults under 30 years of age (albeit only 13% of Class 4) who may have been particularly affected by interruptions to educational courses and graduate employment schemes.^14^ They may therefore have been more vulnerable to changes in employment and financial insecurity brought about by the pandemic, and thus to the subsequent effects on their mental health. It may also be that that the prospect of long-lasting cyclical lockdowns may have posed a particular challenge to this group, with the psychological challenges more about the impact of lockdowns and associated burnout, rather than fear of the virus. Although future health emergencies will undoubtedly follow different patterns, the identification of a group at high risk of deteriorating mental health over time is key to isolating who could benefit from early mental health service intervention to reduce incidence and clinical costs later on.

Another key contribution of this study is identifying factors associated with those who had high levels of distress at the start of the pandemic but soon recovered, compared with those who had high likelihood of symptoms in the clinical range throughout. Those who recovered were on average more likely to be keyworkers and socialise more often before the pandemic. It is possible that these individuals were initially distressed because of strict lockdown measures, the uncertainty of the pandemic, the experience of stressful life events^32^ and, potentially, the added burden or difficulty faced in their work.^33^ However, they appear to have quickly recovered as restrictions eased. This may have been because they were then able to utilise their social support networks or that they were better able to adjust to the ‘new normal’ because of their own resilience or other resources.^5^ Furthermore, some of the initial symptoms captured were likely a result of adjustment problems triggered by the first lockdown, which by definition should abate within 6 months of the stressor being removed (i.e. end of lockdown restrictions). The identification of these positive trajectories after the initial shock is important in showing how some individuals may not need mental health service intervention.

This study is also unique in identifying factors associated with moderate clinically significant symptoms throughout the pandemic compared with emergent clinically significant symptoms. People with emergent clinically significant symptoms were on average less likely to have had a previous mental health diagnosis, and scored marginally lower on openness and neuroticism at the start of the pandemic. This supports the notion that a group of people who did not have clinically significant levels of psychological distress before the pandemic have been disproportionately negatively affected, resulting in the development of incident mental health conditions. The identification of personality-based factors associated with the risk of poorer CMD outcomes during the pandemic can be used to inform formulation-based practice, highlighting individuals based on their personality characteristics who might need further support, or who may be a risk of increasing symptoms.

This study replicated findings in a number of other studies that have suggested that females, younger adults, ^11,12,18^ those with lower incomes and those with physical health concerns have been at increased risk of mental health disorders during the pandemic.^4,11,14,15^ It is worth noting many of these factors had been associated with poorer mental health before the pandemic too.^34–36^ In particular, we found that individuals with previous mental health diagnoses were at higher risk of incident mental health disorders during the study period. This is especially concerning as the reduction in referrals to primary and secondary care mental health services during the pandemic^6,37^ suggests that this group may not have been able to access care as easily, or that they were reluctant to do so, whether because of fears of burdening healthcare systems or some other reason.

### Limitations

The present study used a large data-set, bringing greater precision to previous estimates, and included more recent and frequent data than has been available in other studies. However, there are a number of limitations to note. The raw sample was not fully representative of the UK general adult population. We used survey weights and targeted sampling to improve the representativeness, but these methods cannot remove all sampling biases, and sample weighting could not account for all characteristics that were investigated in this study (e.g. the representativeness of people with a pre-existing mental health diagnosis). Moreover, the sample was limited to people living in England, affecting the wider generalisability of the findings. Additional biases may have been introduced by selecting a minimum of data completed at three time points during the study period as an inclusion criterion. This was selected as it was the minimum requirement for the models fitted, but is likely to have had only a small influence on the results, as the majority of participants provided data for at least 12 time points and sampling weights were generated after sample selection. We chose not to apply corrections for multiple testing in our multinomial regression models, given the exploratory nature of these analyses in identifying potential risk factors, and acknowledge that future studies should consider corrections, especially in more hypothesis-driven research. Residual confounding cannot be ruled out, and we could only explore a range of important covariates available in the data. In particular, depression and anxiety symptom scores before the pandemic were not available. Further detailed information about individualised COVID-19 risk and impact (such as levels of illness, direct impact on family, etc.) were not available, but could be informative about fluctuations in CMD symptom trajectories in future studies. It was decided to model the PHQ-9 and GAD-7 separately in this analysis, as previous analysis using weekly data identified distinct trajectories between the measures;^4^ however, trajectories observed in the current analysis were similar. Analyses modelling simultaneous change in depression and anxiety scores might add further nuance. A further limitation was that self-reported symptom scores were the only measures of CMD symptoms in this study. Other means of identifying symptoms (e.g. via structured clinical interviews) might have provided a somewhat different picture of participants’ CMD symptom experiences,^38^ and studies have found that self-report measures may overestimate the prevalence of mental health issues compared with diagnostic interviews in some circumstances.^24^ However, the PHQ-9 and GAD-7 are widely used in healthcare settings and in epidemiological research, and have been shown to be valid and reliable for identifying clinically meaningful levels of symptoms in various populations.^22,39,40^ Finally, we focused on modelling symptoms that are likely to be clinically significant to inform the future organisation of mental health services, but acknowledge the loss of statistical power from using binary rather than continuous outcomes.

In conclusion, nearly 40% of participants followed trajectories of self-reported depression and anxiety symptoms that were likely to be clinically significant for at least some period of the COVID-19 pandemic. Five distinct trajectories were observed, highlighting the importance of considering heterogenous mental health outcomes rather than population averages. Although three-quarters of individuals showed either consistently high or low incidence of probable mental health disorder, the others showed patterns that fluctuated, either showing early clinically significant symptoms, emergent clinically significant symptoms or fluctuating likelihood of reporting clinically significant symptoms. In future health emergencies, it is important that mental health services differentiate between early and emergent clinically significant symptoms, as although the former are likely to recover on their own within the first few months of the start of an emergency, the latter have a high risk of deterioration over time that could be reduced if appropriate support is provided. The specific predictors of group membership identified in this paper could help inform the development and delivery of public health initiatives in future health emergencies to improve mental well-being, with the potential to prevent the emergence of clinically significant symptoms in a significant portion of the population. It may also support future planning for mental health services, informing estimates of expected levels of need.

## Supporting information

Saunders et al. supplementary material Saunders et al. supplementary material

## Data Availability

The data-sets generated and/or analysed in this study are not publicly available due to stipulations made by the ethics committee. Requests to access the data should be made to the corresponding author, R.S.
