# Supplementary material for: Variation in symptoms of common mental disorders in the general population during the COVID-19 pandemic: longitudinal cohort study
Source: BJPsych Open. 2024 Feb 12;10(2):e45. doi: 10.1192/bjo.2024.2 (PMC10897705; doi:10.1192/bjo.2024.2)
Supplement: Saunders et al. supplementary material [file S2056472424000024sup001.docx]

Supplementary online materials to:

Variation in Symptoms of Common Mental Disorders in the General Population During the COVID-19 Pandemic: A Longitudinal Cohort Study.

**Content:**

Appendix A: COVID-19 Social Study description and participant flow (Page 2)

Appendix B: Measures and participant characteristics (Page 3)

Appendix C: Model selection information (Page 7)

Appendix D: Correspondence between Classes. (Page 9)

Appendix E: Descriptive statistics of trajectories. (Page 11)

Appendix F: Logistic regression analyses. (Page 14)

References (Page 16).

**Appendix A: COVID-19 Social Study description and participant flow.**

Analysis presented in the accompanying manuscript was collected as part of the COVID-19 Social Study. The Study commenced on March 21^st^ 2020, initially as online weekly data collection, which then moved to monthly data collection from August 2020. The study aim was to understand the impact of the Covid-19 on the psychological and social wellbeing of the UK population. The study is not random, and therefore not representation of the UK population but the sample is well-stratified and was recruited through three main approaches. Firstly, convenience sampling was used by promoting the COVID-19 Social Study through existing mailing lists and networks, including a database of adults who have consented to healthcare research previously in the UK, as well as print, digital media coverage and through social media. The second approach was targeted recruitment, focusing on (a) individuals from low-income backgrounds, (b) who were unemployed, and (c) individuals with few educational qualifications. The third approach was promotion through partnerships with third sector organisations to vulnerable participant groups including older people, carers and people with existing mental health conditions. Further detail on recruitment, as well as respondents at each time points are available from the study website: (<https://www.covidsocialstudy.org/>)

From a total of 58,485 participants whose data were available within these dates, 21,051 did not provide data for three time points and were therefore excluded. Of the remaining 37,434, 3731 did not have complete data on predictors including variables for weighting (gender, age, ethnicity, and educational attainment), resulting in a study sample size of 33,703 participants (flow diagram presented in eFigure1 below).

***eFigure1. Participant flow diagram for this study***

**Appendix B: Measures and participant characteristics**

# Measures

## Depression

Depressive symptoms were measured using the Patient Health Questionnaire nine-items (PHQ-9) (Kroenke, Spitzer, & Williams, 2001a); a screening tool for depression used routinely in both clinical and research settings. The nine items of the questionnaire are scored using a 0-3 scale where response options range from “not at all” (0) to “nearly every day” (3). In line with both the developers of the scale and primary care mental health services (Kroenke, Spitzer, & Williams, 2001b; National Collaborating Centre for Mental Health, 2018), all scores were dichotomised into ‘no or mild depression’ (total PHQ-9 scores below 10) and ‘clinically significant depression symptoms’ (scores of 10 or more).

## Anxiety

Generalized anxiety symptoms were measured using the Generalized Anxiety Disorder scale seven-items (GAD-7) (Spitzer, Kroenke, Williams, & Löwe, 2006); a standardised and well-validated screening tool for generalized anxiety disorder used routinely in both clinical and research settings. All seven items are scored using the same response options as those for the PHQ-9. We dichotomised scores into ‘no or mild symptoms’ and ‘clinically significant anxiety symptoms’ using a threshold of ≥8. Whilst the a cut-off of 10 is often used in research settings, a score of 8 or more is used in primary care mental health services such as Improving Access to Psychological Therapies (IAPT) and therefore this lower value was to reflect the level at which is considered ‘cases’ that may warrant formal support in England (NHS, 2018; Saunders et al., 2020).

Details on participant sociodemographic and personality factors collected at study baseline are presented in eTable1 below.

**Supplementary eTable1. Description and categorisation of participant characteristics.**

| **Variable name** | **Description** | **Categories used in analysis** |
| --- | --- | --- |
| Gender | Gender of participant | Grouped in "Women" or "Men" |
| Age | Self-reported age | Four categories: 18-29 year; 30-45 years; 46-59 years; 60+ years old |
| Ethnicity | Self-reported ethnicity | "White" or "Black, Asian and minority ethnic (BAME) groups" |
| Income | Household income | "Low income" defined as <£30,000 per year or "High income" defined as >=£30,000 per year. |
| Education | Education level attained | "General Certificate of Secondary Education (GCSE) or below"; "A-levels or equivalent"; "Undergraduate degree or above" |
| Living situation | Living arrangements with others | "Alone"; "With others including children"; "With others not including any children" |
| Local area | Population density of local area question “What type of area do you currently live? | "Urban"; "Rural" |
| Overcrowded | Whether less than 1 room per person in household | "Yes" or "no" |
| Keyworker | Whether participant's job classed as key or critical worker  Key/critical worker roles are: (1) health, social care or relevant related support worker, (2) teacher or childcare worker still travelling in to work, (3) transport worker still travelling in to work, (4) food chain worker (e.g. production, sale, delivery), (5) key public services worker (e.g. justice staff, religious staff, public service journalist or mortuary worker), (6) local or national government worker delivering essential public services, (7) utility worker (e.g. energy, sewerage, postal service), (8) public safety or national security worker, and (9) worker involved in medicines or protective equipment production or distribution). | "Yes" or "no" |
| Carer | Whether participant has caring responsibilities for elderly relatives or friends, people with long-term conditions or disabilities, or grandchildren. | "Yes" or "no" |
| Mental health condition | Whether participant has previously been diagnosed with a mental health condition | "Yes" or "no" |
| Physical health condition | Whether participant has previously been diagnosed with a physical health condition | "Yes" or "no" |
| Previous amount of social contact | Response to "Usually in your life, how often to meet up with people face to face socially" | Five response categories: "Every day"; "Three or more times a week"; "Once or twice a week"; "Once or twice a month" ;"Less than once a month" |
| Big Five Personality traits | Subscale scores on the BFI-2 | Five subscale scores: "Neuroticism"; "Extraversion"; "Openness"; "Agreeableness"; "Conscientiousness" |

**Retention rate (reproduced from the COVID-19 social Study website: https://www.covidsocialstudy.org/)**

The variable ‘wave’ indicates the sequence of repeated responses for each participant. The baseline response is when ‘wave’ equals 1. As participants could join the study at any point, the response rate across waves is changes daily whilst the study is open. Once study recruitment is complete, we will calculate complete response rates for each wave. As of the 1^st^ November 2021, the number of responses across each wave is as follows. NB the % of participants shown in columns 4 and 5 does not represent a simple retention rate as participants have all joined at different dates across the study period so not everybody has had the ‘opportunity’ to complete multiple waves of data yet. Each week more participants move through the study from previous weeks, increasing the overall numbers who have maintained study engagement.

| **Wave** | **Freq.** | **% of total Obs.** | **% of Wave 1 participants** | **% with Follow-ups** |
| --- | --- | --- | --- | --- |
| **1** | 72,727 | 10.65 | 100 | 79.95 |
| **2** | 58,549 | 8.57 | 80.51 | 87.23 |
| **3** | 50,998 | 7.47 | 70.12 | 89.75 |
| **4** | 45,731 | 6.69 | 62.88 | 91.97 |
| **5** | 41,942 | 6.14 | 57.67 | 92.82 |
| **6** | 39,026 | 5.71 | 53.66 | 93.6 |
| **7** | 36,172 | 5.3 | 49.74 | 95.13 |
| **8** | 35,015 | 5.13 | 48.15 | 95.62 |
| **9** | 33,484 | 4.9 | 46.04 | 96.01 |
| **10** | 32,147 | 4.71 | 44.2 | 96.25 |
| **11** | 30,942 | 4.53 | 42.55 | 96.25 |
| **12** | 29,782 | 4.36 | 40.95 | 95.83 |
| **13** | 28,541 | 4.18 | 39.24 | 95.6 |
| **14** | 27,285 | 3.99 | 37.52 | 93.2 |
| **15** | 25,429 | 3.72 | 34.97 | 90.81 |
| **16** | 23,093 | 3.38 | 31.75 | 90.27 |
| **17** | 20,845 | 3.05 | 28.66 | 87.69 |
| **18** | 18,279 | 2.68 | 25.13 | 80.44 |
| **19** | 14,703 | 2.15 | 20.22 | 69.1 |
| **20** | 10,159 | 1.49 | 13.97 | 63.71 |
| **21** | 6,473 | 0.95 | 8.9 | 27.25 |
| **22** | 1,764 | 0.26 | 2.43 | -- |

NB Wave22 was an incomplete week so participants did not have equal opportunity to participate before the study switched to monthly rather than weekly follow-up.Wave 23 onwards was a monthly wave, consisting of data collected over a period of 4 weeks. As the full sample was re-contacted, the completion rate also increased:

| **Wave** | **Freq.** | **% of total Obs.** | **% of Wave 1 participants** | **% with Follow-ups** |
| --- | --- | --- | --- | --- |
| **23** | 39,619 | 8.62 | 100 | 92.5 |
| **24** | 33,736 | 7.34 | 85.15 | 96.5 |
| **25** | 30,846 | 6.71 | 77.86 | 97.48 |
| **26** | 28,664 | 6.24 | 72.35 | 98.31 |
| **27** | 27,208 | 5.92 | 68.67 | 98.9 |
| **28** | 26,300 | 5.72 | 66.38 | 98.79 |
| **29** | 25,380 | 5.52 | 64.06 | 98.19 |
| **30** | 36,579 | 7.96 | 92.33 | 88.33 |
| **31** | 30,215 | 6.58 | 76.26 | 94.16 |
| **32** | 27,111 | 5.9 | 68.43 | 96.07 |
| **33** | 24,893 | 5.42 | 62.83 | 97.85 |
| **34** | 23,564 | 5.13 | 59.48 | 97.82 |
| **35** | 22,213 | 4.84 | 56.07 | 98.27 |
| **36** | 21,132 | 4.6 | 53.34 | 98.41 |
| **37** | 19,703 | 4.29 | 49.73 | 97.29 |
| **38** | 9,919 | 2.16 | 25.04 | 96.84 |
| **39** | 32,317 | 7.03 | 81.57 | -- |

**Appendix C: Model selection information**

We used established model fit criteria to identify the optimal growth mixture model solution (Nylund, Asparouhov, & Muthén, 2007). Specifically, the Vuong-Lo-Medell-Rubin Likelihood Ratio Test (VLMR-LRT) (Lo, Mendell, & Rubin, 2001), the Akaike Information Criterion (AIC), Bayesian Information Criterion (BIC), and entropy value were considered between class solutions. The VLMR-LRT compares a model with K-classes against the K-1 model, with a p-value < 0.05 indicating that the K model is a better fit to the data than the K-1 model. Models with lower AIC and BIC values are preferred and higher entropy values indicate better classification accuracy (Geiser, 2013; Saunders et al., 2019). We assumed no prior hypotheses about the anticipated number of classes and therefore conducted GMM first with a two-class model (identifying two classes) and then increase the number of classes by one each time until the VLMR-LRT became non-significant (p>0.05) or either of the AIC or BIC values increased when compared to the previous K-1 class, in line with established guidance and previous research employing technique (Musliner et al., 2016; Nylund et al., 2007; Saunders et al., 2019).

We present model fit statistics for the current analyses in eTable2 below. For both the PHQ-9 and GAD-7 analyses, the AIC and BIC values were observed to decrease as the number of classes increased, however the VLMR-LRT indicated that there was little evidence the 6-class solution provided a better fit than the 5-class model for both measures. The 5-class model was selected for both measures, and each participant was allocated to the profile to which they had the highest probability of membership.

**Supplementary eTable2. Model fit statistics for GAD-7 and PHQ-9 GMMs.**

| **Anxiety symptoms (GAD-7)** | | | | | | | |
| --- | --- | --- | --- | --- | --- | --- | --- |
| **Class Solution** | **Log-Likelihood** | **AIC** | **BIC** | **Adj-BIC** | **VLMR-LRT p-value** | **Entropy** | **% individuals per class** |
| 2-Class | -96481 | 192997 | 193140 | 193086 | <0.001 | 0.928 | 74/26 |
| 3-Class | -90162 | 180364 | 180532 | 180469 | <0.001 | 0.848 | 16/64/20 |
| 4-Class | -89339 | 178725 | 178919 | 178846 | <0.001 | 0.779 | 13/16/61/11 |
| **5-Class** | **-88775** | **177602** | **177821** | **177739** | **<0.001** | **0.79** | **6/9/13/63/9** |
| 6-Class | -88485 | 177029 | 177273 | 177181 | 0.240 | 0.779 | 62/13/9/6/5/5 |
| **Depression symptoms (PHQ-9)** | | | | | | | |
| **Class Solution** | **Log-Likelihood** | **AIC** | **BIC** | **Adj-BIC** | **VLMR-LRT p-value** | **Entropy** | **% individuals per class** |
| 2-Class | -98812 | 197657 | 197800 | 197746 | <0.001 | 0.927 | 27/72 |
| 3-Class | -92243 | 184527 | 184696 | 184632 | <0.001 | 0.848 | 16/63/20 |
| 4-Class | -91455 | 182957 | 183151 | 183077 | <0.001 | 0.775 | 11/13/60/15 |
| **5-Class** | **-90797** | **181646** | **181865** | **181783** | **<0.001** | **0.79** | **6/62/13/10/9** |
| 6-Class | -90482 | 181023 | 181267 | 181175 | 0.178 | 0.784 | 61/13/10/7/5/4 |

**Appendix D: Correspondence between Classes**

The proportion of individuals reporting being in the clinically-significant range of the PHQ-9 and GAD-7 and each timepoint is presented in eTable6 and eTable7 shows the correspondence between individuals being anxiety and depression trajectory classes. Whilst there was higher correspondence between individuals between in Classes 1 and 2, for Classes 3, 4 and 5 there was more variation as to whether individuals were members of the corresponding class for each measure.

**eTable 3. Correspondence between PHQ-9 and GAD-7 caseness at each timepoint**

|  |  | **%: GAD-7 caseness=0** | **%: GAD-7 caseness=1** |
| --- | --- | --- | --- |
| **Mar-20** | **%: PHQ-9 caseness = 0** | 88.99% | 11.01% |
|  | **%: PHQ-9 caseness = 1** | 22.94% | 77.06% |
| **Apr-20** | **%: PHQ-9 caseness = 0** | 93.21% | 6.79% |
|  | **%: PHQ-9 caseness = 1** | 25.85% | 74.15% |
| **May-20** | **%: PHQ-9 caseness = 0** | 94.33% | 5.67% |
|  | **%: PHQ-9 caseness = 1** | 26.17% | 73.83% |
| **Jun-20** | **%: PHQ-9 caseness = 0** | 94.95% | 5.05% |
|  | **%: PHQ-9 caseness = 1** | 26.18% | 73.82% |
| **Jul-20** | **%: PHQ-9 caseness = 0** | 95.51% | 4.49% |
|  | **%: PHQ-9 caseness = 1** | 26.36% | 73.64% |
| **Aug-20** | **%: PHQ-9 caseness = 0** | 93.41% | 6.59% |
|  | **%: PHQ-9 caseness = 1** | 26.14% | 73.86% |
| **Sep-20** | **%: PHQ-9 caseness = 0** | 93.05% | 6.95% |
|  | **%: PHQ-9 caseness = 1** | 26.34% | 73.66% |
| **Oct-20** | **%: PHQ-9 caseness = 0** | 93.15% | 6.85% |
|  | **%: PHQ-9 caseness = 1** | 28.48% | 71.52% |
| **Nov-20** | **%: PHQ-9 caseness = 0** | 93.60% | 6.40% |
|  | **%: PHQ-9 caseness = 1** | 27.95% | 72.05% |
| **Dec-20** | **%: PHQ-9 caseness = 0** | 93.57% | 6.43% |
|  | **%: PHQ-9 caseness = 1** | 27.08% | 72.92% |
| **Jan-21** | **%: PHQ-9 caseness = 0** | 94.24% | 5.76% |
|  | **%: PHQ-9 caseness = 1** | 29.32% | 70.68% |
| **Feb-21** | **%: PHQ-9 caseness = 0** | 94.30% | 5.70% |
|  | **%: PHQ-9 caseness = 1** | 30.31% | 69.69% |
| **Mar-21** | **%: PHQ-9 caseness = 0** | 93.98% | 6.02% |
|  | **%: PHQ-9 caseness = 1** | 27.55% | 72.45% |
| **Apr-21** | **%: PHQ-9 caseness = 0** | 93.85% | 6.15% |
|  | **%: PHQ-9 caseness = 1** | 29.13% | 70.87% |

**eTable 4. Correspondence between PHQ-9 and GAD-7 Classes (Weighted sample)**

|  | GAD-7 Class | | | | | | | | | |  |
| --- | --- | --- | --- | --- | --- | --- | --- | --- | --- | --- | --- |
| PHQ Class | 1 | | 2 | | 3 | | 4 | | 5 | | Total |
|  | n | % | n | % | n | % | n | % | n | % |  |
| 1 | 18630 | 88.0% | 106 | 2.5% | 1054 | 34.6% | 673 | 31.9% | 354 | 11.2% | 20818 |
| 2 | 226 | 1.1% | 3100 | 73.6% | 209 | 6.9% | 177 | 8.4% | 731 | 23.1% | 4444 |
| 3 | 957 | 4.5% | 215 | 5.1% | 1218 | 39.9% | 122 | 5.8% | 540 | 17.1% | 3053 |
| 4 | 830 | 3.9% | 109 | 2.6% | 167 | 5.5% | 757 | 35.9% | 294 | 9.3% | 2158 |
| 5 | 523 | 2.5% | 683 | 16.2% | 402 | 13.2% | 382 | 18.1% | 1240 | 39.2% | 3230 |
| Total | 21167 |  | 4214 |  | 3051 |  | 2111 |  | 3160 |  | 33703 |

**Appendix E: Descriptive statistics of trajectories.**

**Supplementary eTable5. Descriptive statistics of the identified GAD-7 and PHQ-9 classes.**

|  | **GAD-7 classes** | | | | | **PHQ-9 Classes** | | | | |
| --- | --- | --- | --- | --- | --- | --- | --- | --- | --- | --- |
|  | **Class 1**  **(n=20818)** | **Class 2**  **(n=4444)** | **Class 3**  **(n=3053)** | **Class 4**  **(n=2158)** | **Class 5**  **(n=3230)** | **Class 1**  **(n=21167)** | **Class 2**  **(n=4214)** | **Class 3**  **(n=3051)** | **Class 4**  **(n=2111)** | **Class 5**  **(n=3160)** |
|  | **N (%)** | **N (%)** | **N (%)** | **N (%)** | **N (%)** | **N (%)** | **N (%)** | **N (%)** | **N (%)** | **N (%)** |
| **Gender** | | | | | | | | | | |
| Women | 9698(46) | 1232(29) | 758(25) | 666(32) | 860(27) | 9343(45) | 1354(30) | 819(27) | 716(33) | 982(30) |
| Men | 11469(54) | 2983(71) | 2293(75) | 1445(68) | 2300(73) | 11475(55) | 3090(70) | 2234(73) | 1442(67) | 2249(70) |
| **Age in years** | | | | | | | | | | |
| 18-29 | 1798(9) | 742(18) | 501(16) | 275(13) | 569(18) | 1890(9) | 707(16) | 540(18) | 243(11) | 505(16) |
| 30-45 | 6921(33) | 1778(42) | 1301(43) | 847(40) | 1311(41) | 7010(34) | 1731(39) | 1249(41) | 858(40) | 1309(41) |
| 46-59 | 7595(36) | 1341(32) | 1004(33) | 706(33) | 976(31) | 7285(35) | 1554(35) | 1008(33) | 737(34) | 1036(32) |
| 60+ | 4853(23) | 353(8) | 244(8) | 284(13) | 304(10) | 4632(22) | 452(10) | 255(8) | 319(15) | 380(12) |
| **Ethnicity** | | | | | | | | | | |
| White | 19606(93) | 3831(91) | 2777(91) | 1895(90) | 2817(89) | 19234(92) | 4033(91) | 2773(91) | 1987(92) | 2900(90) |
| BAME | 1561(7) | 384(9) | 274(9) | 216(10) | 343(11) | 1584(8) | 411(9) | 280(9) | 171(8) | 331(10) |
| **Household income** | | | | | | | | | | |
| <£30,000 | 13665(65) | 1881(45) | 1886(62) | 1294(61) | 1787(57) | 13851(67) | 1751(39) | 1811(59) | 1314(61) | 1786(55) |
| >=£30,000 | 7502(35) | 2333(55) | 1165(38) | 817(39) | 1372(43) | 6967(33) | 2693(61) | 1242(41) | 843(39) | 1445(45) |
| **Keyworker** | | | | | | | | | | |
| No | 15958(75) | 3346(79) | 2150(70) | 1562(74) | 2373(75) | 15644(75) | 3549(80) | 2207(72) | 1586(73) | 2404(74) |
| Yes | 5209(25) | 869(21) | 900(30) | 549(26) | 787(25) | 5174(25) | 895(20) | 845(28) | 572(27) | 827(26) |
| **Education** | | | | | | | | | | |
| GCSE or below | 5113(24) | 1145(27) | 687(23) | 407(19) | 706(22) | 4840(23) | 1296(29) | 687(23) | 503(23) | 731(23) |
| A-levels or equivalent | 5571(26) | 1327(31) | 826(27) | 591(28) | 861(27) | 5361(26) | 1493(34) | 879(29) | 574(27) | 869(27) |
| Degree or above | 10484(50) | 1743(41) | 1538(50) | 1113(53) | 1592(50) | 10618(51) | 1654(37) | 1487(49) | 1081(50) | 1630(50) |
| **Carer** | | | | | | | | | | |
| No | 18250(86) | 3509(83) | 2527(83) | 1813(86) | 2668(84) | 17988(86) | 3680(83) | 2565(84) | 1794(83) | 2740(85) |
| Yes | 2917(14) | 705(17) | 524(17) | 298(14) | 491(16) | 2830(14) | 764(17) | 488(16) | 363(17) | 491(15) |
| **Living situation** | | | | | | | | | | |
| Alone | 3673(17) | 853(20) | 451(15) | 361(17) | 507(16) | 3303(16) | 1080(24) | 507(17) | 354(16) | 602(19) |
| With others, no children | 11392(54) | 2050(49) | 1505(49) | 1106(52) | 1619(51) | 11376(55) | 2034(46) | 1458(48) | 1113(52) | 1691(52) |
| With others, with children | 6102(29) | 1312(31) | 1094(36) | 645(31) | 1033(33) | 6139(29) | 1330(30) | 1089(36) | 691(32) | 937(29) |
| **Overcrowded** | | | | | | | | | | |
| No | 18783(89) | 3342(79) | 2513(82) | 1786(85) | 2581(82) | 18422(88) | 3589(81) | 2458(81) | 1852(86) | 2683(83) |
| Yes | 2384(11) | 873(21) | 538(18) | 325(15) | 579(18) | 2396(12) | 855(19) | 595(19) | 305(14) | 547(17) |
| **Urban/Rural** | | | | | | | | | | |
| Rural | 16844(80) | 3506(83) | 2514(82) | 1753(83) | 2630(83) | 16605(80) | 3696(83) | 2481(81) | 1742(81) | 2723(84) |
| Urban | 4323(20) | 708(17) | 537(18) | 359(17) | 530(17) | 4213(20) | 748(17) | 572(19) | 415(19) | 508(16) |
| **Diagnosed mental illness** | | | | | | | | | | |
| No | 19299(91) | 1819(43) | 2124(70) | 1651(78) | 1931(61) | 19107(92) | 1852(42) | 2082(68) | 1735(80) | 2048(63) |
| Yes | 1868(9) | 2395(57) | 927(30) | 461(22) | 1228(39) | 1711(8) | 2593(58) | 971(32) | 423(20) | 1182(37) |
| **Long-term physical health condition** | | | | | | | | | | |
| No | 13947(66) | 2079(49) | 1894(62) | 1298(61) | 1829(58) | 14014(67) | 1971(44) | 1904(62) | 1321(61) | 1837(57) |
| Yes | 7220(34) | 2135(51) | 1157(38) | 813(39) | 1331(42) | 6804(33) | 2473(56) | 1149(38) | 837(39) | 1393(43) |
| **Previous social contact frequency** | | | | | | | | | | |
| Every day | 1950(9) | 409(10) | 329(11) | 184(9) | 310(10) | 1927(9) | 427(10) | 311(10) | 215(10) | 301(9) |
| Three or more times a week | 4911(23) | 792(19) | 679(22) | 457(22) | 633(20) | 4896(24) | 781(18) | 677(22) | 483(22) | 635(20) |
| Once or twice a week | 7654(36) | 1326(31) | 1070(35) | 743(35) | 1110(35) | 7594(36) | 1346(30) | 1111(36) | 734(34) | 1119(35) |
| Once or twice a month | 4364(21) | 834(20) | 606(20) | 453(21) | 693(22) | 4245(20) | 920(21) | 601(20) | 490(23) | 695(22) |
| Less than once a month | 2288(11) | 853(20) | 368(12) | 274(13) | 414(13) | 2156(10) | 970(22) | 354(12) | 236(11) | 481(15) |
| **Big Five personality factor** | **M (SD)** | **M (SD)** | **M (SD)** | **M (SD)** | **M (SD)** | **M (SD)** | **M (SD)** | **M (SD)** | **M (SD)** | **M (SD)** |
| Neuroticism | 10.1(3.9) | 15.8(3.6) | 13.9(3.6) | 11.8(3.8) | 14.1(3.8) | 10.3(4) | 15.1(4) | 13.5(4) | 12(4) | 13(4) |
| Extraversion | 12.8(4.2) | 11.4(4.5) | 12.6(4.4) | 12.7(4.4) | 12.1(4.4) | 12.9(4.2) | 11.2(4.4) | 12.5(4.4) | 13(4) | 12(4) |
| Openness | 14.9(3.2) | 15.2(3.8) | 15.3(3.3) | 15.1(3.3) | 15.4(3.5) | 14.9(3.2) | 15(3.7) | 15.2(3.3) | 15(3) | 15(3) |
| Agreeableness | 15.5(3) | 15.3(3.5) | 15.6(3.1) | 15.5(3.2) | 15.5(3.3) | 15.5(3) | 15.3(3.5) | 15.4(3.1) | 15(3) | 15(3) |
| Conscientiousness | 15.8(2.9) | 15.2(3.3) | 15.8(3.1) | 15.6(2.9) | 15.5(3.2) | 16(2.8) | 15(3.4) | 15.4(3.1) | 16(3) | 15(3) |

**Appendix F: Logistic regression analyses.**

**Supplementary eTable6.**

**Comparison of Class 3 with Class 2 (Logistic regression).**

|  | **PHQ: Class 3 (vs Class 2)** | | |  | **GAD: Class 3 (vs Class 2)** | | |
| --- | --- | --- | --- | --- | --- | --- | --- |
|  | **OR** | ***95% CIs*** | **p-value** |  | **OR** | ***95% CIs*** | **p-value** |
| **Gender: Women (vs men)** | **1.26** | ***(1.06;1.49)*** | **0.008** |  | **1.41** | ***(1.19;1.67)*** | **<0.001** |
| **Age: 18-29 years (vs 60+ years)** | 1.03 | *(0.78;1.36)* | 0.834 |  | 0.86 | *(0.65;1.13)* | 0.281 |
| **Age: 30 to 45 years (vs 60+ years)** | 1.01 | *(0.79;1.29)* | 0.935 |  | 0.90 | *(0.71;1.15)* | 0.404 |
| **Age: 46 to 59 years (vs 60+ years)** | 1.08 | *(0.86;1.35)* | 0.507 |  | 1.03 | *(0.83;1.29)* | 0.770 |
| **Ethnicity: Black, Asian, Minority (vs White)** | **0.73** | ***(0.56;0.94)*** | **0.014** |  | 0.85 | *(0.66;1.09)* | 0.201 |
| **Education: Low (vs High)** | **0.81** | ***(0.67;0.99)*** | **0.039** |  | 0.88 | *(0.72;1.08)* | 0.219 |
| **Education: Medium (vs High)** | **0.79** | ***(0.67;0.93)*** | **0.006** |  | 0.85 | *(0.71;1)* | 0.052 |
| **Income: <£30,000 (vs >£30,000)** | **0.65** | ***(0.56;0.76)*** | **<0.001** |  | **0.70** | ***(0.6;0.82)*** | **<0.001** |
| **Alone (vs With others, no children)** | 0.83 | *(0.69;1)* | 0.051 |  | 0.89 | *(0.73;1.07)* | 0.214 |
| **Living with others, with children (vs Others, no children)** | 1.10 | *(0.93;1.29)* | 0.269 |  | 1.09 | *(0.93;1.27)* | 0.304 |
| **Mental health diagnosis (vs none)** | **0.46** | ***(0.4;0.53)*** | **<0.001** |  | **0.49** | ***(0.42;0.57)*** | **<0.001** |
| **Carer (vs not a carer)** | **0.82** | ***(0.69;0.99)*** | **0.034** |  | 0.92 | *(0.77;1.09)* | 0.342 |
| **Keyworker (vs not a keyworker)** | **1.20** | ***(1.02;1.41)*** | **0.025** |  | **1.25** | ***(1.07;1.46)*** | **0.006** |
| **Long-term health condition (vs none)** | **0.66** | ***(0.57;0.76)*** | **<0.001** |  | **0.77** | ***(0.67;0.89)*** | **<0.001** |
| **Overcrowded living (vs not)** | 1.07 | *(0.89;1.3)* | 0.454 |  | 0.88 | *(0.73;1.06)* | 0.174 |
| **Urban (vs Rural)** | 1.16 | *(0.97;1.38)* | 0.098 |  | 0.99 | *(0.83;1.18)* | 0.900 |
| **Social: every day (vs once/twice a week)** | **0.77** | ***(0.6;0.99)*** | **0.039** |  | 0.94 | *(0.73;1.2)* | 0.614 |
| **Social: three/four times a week (vs once/twice a week)** | 0.95 | *(0.79;1.14)* | 0.568 |  | 0.98 | *(0.82;1.17)* | 0.807 |
| **Social: once/twice a month (vs once/twice a week)** | **0.76** | ***(0.63;0.92)*** | **0.004** |  | 0.92 | *(0.76;1.11)* | 0.389 |
| **Social: less once month (vs once/twice a week)** | **0.59** | ***(0.47;0.73)*** | **<0.001** |  | **0.73** | ***(0.59;0.91)*** | **0.006** |
| **Personality: Neuroticism** | **0.94** | ***(0.93;0.96)*** | **<0.001** |  | **0.90** | ***(0.88;0.92)*** | **<0.001** |
| **Personality: Extraversion** | **1.03** | ***(1.01;1.05)*** | **<0.001** |  | **1.02** | ***(1.01;1.04)*** | **0.006** |
| **Personality: Openness** | 0.99 | *(0.97;1.01)* | 0.208 |  | **0.98** | ***(0.96;1)*** | **0.029** |
| **Personality: Agreeableness** | 1.00 | *(0.98;1.02)* | 0.977 |  | 1.01 | *(0.99;1.03)* | 0.257 |
| **Personality: Conscientiousness** | 1.01 | *(0.99;1.03)* | 0.395 |  | 1.00 | *(0.98;1.03)* | 0.682 |
| Notes: OR = Odds ratio; 95%CIs = 95% confidence intervals | |  |  |  |  |  |  |

**Supplementary eTable7.**

**Comparison of Class 5 with Class 4 (Logistic regression).**

|  | **PHQ: Class 5 (vs Class 4)** | | |  | **GAD: Class 5 (vs Class 4)** | | |
| --- | --- | --- | --- | --- | --- | --- | --- |
|  | **OR** | ***95% CIs*** | **p-value** |  | **OR** | ***95% CIs*** | **p-value** |
| **Gender: Women (vs men)** | 1.01 | *(0.85;1.21)* | 0.872 |  | 0.96 | *(0.8;1.16)* | 0.659 |
| **Age: 18-29 years (vs 60+ years)** | **1.39** | ***(1.02;1.89)*** | **0.039** |  | **1.82** | ***(1.32;2.51)*** | **<0.001** |
| **Age: 30 to 45 years (vs 60+ years)** | 1.21 | *(0.94;1.54)* | 0.139 |  | **1.47** | ***(1.13;1.92)*** | **0.004** |
| **Age: 46 to 59 years (vs 60+ years)** | 1.12 | *(0.89;1.41)* | 0.321 |  | 1.22 | *(0.95;1.56)* | 0.117 |
| **Ethnicity: Black, Asian, Minority (vs White)** | 1.28 | *(0.96;1.71)* | 0.088 |  | 1.18 | *(0.89;1.55)* | 0.244 |
| **Education: Low (vs High)** | 0.99 | *(0.79;1.24)* | 0.908 |  | **1.46** | ***(1.15;1.85)*** | **0.002** |
| **Education: Medium (vs High)** | 1.00 | *(0.83;1.21)* | 0.988 |  | 0.95 | *(0.79;1.15)* | 0.600 |
| **Income: <£30,000 (vs >£30,000)** | 1.14 | *(0.96;1.36)* | 0.121 |  | 1.11 | *(0.93;1.33)* | 0.237 |
| **Alone (vs With others, no children)** | 1.13 | *(0.92;1.39)* | 0.235 |  | 0.94 | *(0.76;1.17)* | 0.582 |
| **Living with others, with children (vs Others, no children)** | 0.91 | *(0.76;1.09)* | 0.303 |  | 1.17 | *(0.98;1.4)* | 0.086 |
| **Mental health diagnosis (vs none)** | **1.86** | ***(1.56;2.22)*** | **<0.001** |  | **1.70** | ***(1.42;2.03)*** | **<0.001** |
| **Carer (vs not a carer)** | 0.95 | *(0.78;1.16)* | 0.606 |  | **1.25** | ***(1.01;1.55)*** | **0.039** |
| **Keyworker (vs not a keyworker)** | 1.08 | *(0.91;1.28)* | 0.394 |  | 1.02 | *(0.85;1.21)* | 0.859 |
| **Long-term health condition (vs none)** | **1.18** | ***(1.01;1.38)*** | **0.033** |  | 1.14 | *(0.97;1.35)* | 0.113 |
| **Overcrowded living (vs not)** | 1.16 | *(0.92;1.45)* | 0.201 |  | 1.07 | *(0.85;1.33)* | 0.575 |
| **Urban (vs Rural)** | 0.87 | *(0.72;1.05)* | 0.142 |  | 1.07 | *(0.88;1.31)* | 0.496 |
| **Social: every day (vs once/twice a week)** | 1.01 | *(0.77;1.31)* | 0.956 |  | **1.36** | ***(1.01;1.82)*** | **0.039** |
| **Social: three/four times a week (vs once/twice a week)** | 0.90 | *(0.74;1.1)* | 0.310 |  | 1.01 | *(0.82;1.23)* | 0.951 |
| **Social: once/twice a month (vs once/twice a week)** | 0.92 | *(0.75;1.13)* | 0.424 |  | 1.01 | *(0.83;1.24)* | 0.906 |
| **Social: less once month (vs once/twice a week)** | **1.37** | ***(1.06;1.78)*** | **0.018** |  | 0.97 | *(0.74;1.27)* | 0.831 |
| **Personality: Neuroticism** | **1.08** | ***(1.06;1.1)*** | **<0.001** |  | **1.16** | ***(1.13;1.18)*** | **<0.001** |
| **Personality: Extraversion** | 1.00 | *(0.98;1.02)* | 0.953 |  | 0.99 | *(0.97;1.01)* | 0.511 |
| **Personality: Openness** | **1.02** | ***(1;1.05)*** | **0.045** |  | **1.06** | ***(1.04;1.09)*** | **<0.001** |
| **Personality: Agreeableness** | 1.00 | *(0.97;1.02)* | 0.874 |  | 0.99 | *(0.97;1.02)* | 0.634 |
| **Personality: Conscientiousness** | **0.97** | ***(0.95;1)*** | **0.021** |  | 1.01 | *(0.98;1.03)* | 0.663 |
| Notes: OR = Odds ratio; 95%CIs = 95% confidence intervals | |  |  |  |  |  |  |

**References**

Geiser, C. (2013). *Data analysis with Mplus*. New York: Guilford.

Kroenke, K., Spitzer, R. L., & Williams, J. B. W. (2001a). The PHQ-9: Validity of a Brief Depression Severity Measure. *Journal of General Internal Medicine*, *16*, 606–613.

Kroenke, K., Spitzer, R. L., & Williams, J. B. W. (2001b). The PHQ-9. *Journal of General Internal Medicine*, *16*(9), 606–613. https://doi.org/10.1046/j.1525-1497.2001.016009606.x

Lo, Y., Mendell, N. R., & Rubin, D. B. (2001). Testing the number of components in a normal mixture. *Biometrika*, *88*(3), 767–778.

Musliner, K. L., Munk-Olsen, T., Laursen, T. M., Eaton, W. W., Zandi, P. P., & Mortensen, P. B. (2016). Heterogeneity in 10-Year Course Trajectories of Moderate to Severe Major Depressive Disorder. *JAMA Psychiatry*, *73*(4), 346. https://doi.org/10.1001/jamapsychiatry.2015.3365

National Collaborating Centre for Mental Health. (2018). The Improving Access to Psychological Therapies Manual. *NHS Digital*.

NHS. (2018). *Psychological Therapies, Annual Report on the use of IAPT Services*. *NHS Digital*. London.

Nylund, K. L., Asparouhov, T., & Muthén, B. O. (2007). Deciding on the number of classes in latent class analysis and growth mixture modeling: A Monte Carlo simulation study. *Structural Equation Modeling*, *14*(4), 535–569. https://doi.org/10.1080/10705510701575396

Saunders, R., Buckman, J. E. J., Cape, J., Fearon, P., Leibowitz, J., & Pilling, S. (2019). Trajectories of depression and anxiety symptom change during psychological therapy. *Journal of Affective Disorders*, *249*, 327–335. https://doi.org/10.1016/J.JAD.2019.02.043

Saunders, R., Cape, J., Leibowitz, J., Aguirre, E., Jena, R., Cirkovic, M., … Buckman, J. E. J. (2020). Improvement in IAPT outcomes over time: Are they driven by changes in clinical practice? *Cognitive Behaviour Therapist*, *13*, e16. https://doi.org/10.1017/S1754470X20000173

Spitzer, R. L., Kroenke, K., Williams, J. B. W., & Löwe, B. (2006). A Brief Measure for Assessing Generalized Anxiety Disorder. *Archives of Internal Medicine*, *166*(10), 1092. https://doi.org/10.1001/archinte.166.10.1092
